# How Much Do We Know about the Biopsychosocial Predictors of Glycaemic Control? Age and Clinical Factors Predict Glycaemic Control, but Psychological Factors Do Not

**DOI:** 10.1155/2020/2654208

**Published:** 2020-05-01

**Authors:** Mohammad Farris Iman Leong Bin Abdullah, Hatta Sidi, Arun Ravindran, Paula Junggar Gosse, Emily Samantha Kaunismaa, Roslyn Laurie Mainland, Norlaila Mustafa, Nurul Hazwani Hatta, Puteri Arnawati, Amelia Yasmin Zulkifli, Luke Sy-Cherng Woon

**Affiliations:** ^1^Advanced Medical and Dental Institute, Universiti Sains Malaysia, Malaysia; ^2^Department of Psychiatry, Universiti Kebangsaan Malaysia Medical Centre, Malaysia; ^3^Centre for Addiction and Mental Health, University of Toronto, Canada; ^4^Faculty of Medicine, University of Toronto, Canada; ^5^Department of Medicine, Universiti Kebangsaan Malaysia Medical Centre, Malaysia; ^6^University of Galway, Ireland

## Abstract

**Objective:**

Diabetes mellitus is one of the most common noncommunicable diseases in Malaysia. It is associated with significant complications and a high cost of treatment, especially when glycaemic control is poor. Despite its negative impact on health, data is still lacking on the possible biopsychosocial predictors of poor glycaemic control among the diabetic population. This study is aimed at determining the prevalence of poor glycaemic control as well as its association with biopsychosocial factors such as personality traits, psychiatric factors, and quality of life (QOL) among Malaysian patients with diabetes.

**Methods:**

A cross-sectional study was conducted at the Universiti Kebangsaan Malaysia Medical Centre (UKMMC) using outpatient population diabetic patients. Demographic data on social and clinical characteristics were collected from participants. Several questionnaires were administered, including the Beck Depression Inventory-II (BDI-II) to measure depressive symptoms, the Generalized Anxiety Disorder-7 (GAD-7) to assess anxiety symptoms, the Big Five Inventory (BFI) to evaluate personality traits, and the WHO Quality of Life-BREF (WHOQOL-BREF) to assess QOL. Multivariate binary logistic regression was performed to determine the predictors of poor glycaemic control.

**Results:**

300 patients with diabetes mellitus were recruited, with the majority (90%) having type 2 diabetes. In this population, the prevalence of poor glycaemic control (HbA_1C_ ≥ 7.0%) was 69%, with a median HbA_1C_ of 7.6% (IQR = 2.7). Longer duration of diabetes mellitus and a greater number of days of missed medications predicted poor glycaemic control, while older age and overall self-perception of QOL protected against poor glycaemic control. No psychological factors were associated with poor glycaemic control.

**Conclusion:**

This study emphasizes the importance of considering the various factors that contribute to poor glycaemic control, such as duration of diabetes, medication adherence, age, and QOL. These findings should be used by clinicians, particularly when planning a multidisciplinary approach to the management of diabetes.

## 1. Introduction

Diabetes mellitus is one of the most common chronic diseases worldwide, affecting 422 million people in 2014. It is expected to increase in prevalence, with an estimated 629 million people affected by 2045 [1]. While diabetes is a worldwide health crisis, the prevalence of diabetes is increasing at a more rapid rate in lower- and middle-income countries [2]. Patients in these countries often have limited access to healthcare resources, potentially exacerbating both their diabetes and its associated comorbidities [3]. Furthermore, much of the research on diabetes as it relates to biopsychosocial factors has been conducted in higher-income countries, despite the strong need to understand these relationships in lower- and middle-income countries where individuals often face significant barriers to care. As such, this study was conducted in Malaysia, a middle-income country where the estimated prevalence of diabetes is 17.5% [2], over 10% higher than the global average [4].

In Malaysia, diabetes mellitus is currently the second most common noncommunicable disease. The prevalence of diabetes increases with age, ranging from 5.5% in the 18–19-year age group to 39.1% in the 70–74-year age group [5]. In 2011, the estimated cost of managing diabetes in the country was 2.04 billion Malaysian ringgits (493 million USD), while the estimated cost per patient was approximately RM 68,000 (16,000 USD) [6]. Hence, there is significant economic burden associated with the management of diabetes mellitus in Malaysia.

Diabetic complications can be divided into macrovascular and microvascular complications which worsen over time if optimal glycaemic control is not achieved. Among the diabetic population in Malaysia, microvascular complications, such as nephropathy (7.8%) and retinopathy (6.7%), are common. Macrovascular complications are less common and include ischaemic heart disease (5.3%), cerebrovascular incidents (1.3%), and diabetic foot ulcers (1.2%) which can lead to amputations (0.9%). According to the 2009–2012 National Diabetes Registry of Malaysia, diabetic complications are underestimated due to a high proportion of missing data [7]. In fact, a recent review of Malaysian statistics on diabetes suggests that the true prevalence of microvascular complications is closer to 75% and macrovascular complications is closer to 29% [8]. It is therefore important to explore factors that predict glycaemic control so that treatment strategies can be designed to effectively manage diabetes and its complications.

In the context of the biopsychosocial model, data is lacking on the predictors of glycaemic control in the Malaysian population. Only a few previous studies on Malaysian patients with diabetes have reported associations between biopsychosocial factors and poor glycaemic control. These studies reported that the following factors are significantly associated with poor glycaemic control: older age (60–69 years old), being identified as Malay or Indian, having a duration of diabetes ≥ 5 years, the use of oral hypoglycaemic agents and insulin, comorbid hypertension and hyperlipidaemia, and patient perception that diabetes interferes with daily activities. Being unemployed or retired and having up to tertiary education are all protective factors against poor glycaemic control. Psychiatric disorders, such as depression and anxiety, have not been found to be predictive of glycaemic control in Malaysian diabetic patients [9–11]. However, personality traits, such as type D personality (social inhibition, negative affectivity, and neuroticism), have previously been associated with diabetes mellitus [12]. As far as we are aware, no study has assessed the relationships between personality traits, quality of life (QOL), and glycaemic control in Malaysian patients with diabetes. To address this research gap, the Anxiety, Depression, and Personality Traits in Diabetes Mellitus (ADAPT-DM) study is aimed at determining the prevalence of poor glycaemic control as well as its relationship with biopsychosocial factors, including personality traits, psychiatric factors, and QOL among diabetic patients.

## 2. Materials and Methods

### 2.1. Study Participants and Setting

The ADAPT-DM study was a cross-sectional study which recruited patients who were diagnosed with diabetes mellitus. The study was approved by the Medical Research Committee of the Faculty of Medicine, Universiti Kebangsaan Malaysia (UKM FPR.SPI 800-2/28/166/FF-2019-342). The participants were recruited via convenience sampling from the outpatient endocrine clinic of Universiti Kebangsaan Malaysia Medical Centre (UKMMC). Patients were eligible if they were at least 18 years old and had a known diagnosis of diabetes mellitus (type 1, type 2, or gestational diabetes mellitus). Patients were excluded if they presented with cognitive impairment or with psychotic symptoms. During the two-month enrolment period, patients who met the eligibility criteria were invited to participate in the study. Patients were presented with a consent form and an oral briefing in their preferred language (Malay or English). Information that was provided included study objectives, length, risks and benefits of participation, measures to maintain confidentiality, and participant rights and responsibilities. Respondents who provided written consent to participate were asked to complete all components of the study to ensure study quality.

### 2.2. Data Collection

Participants were asked to complete six standardized questionnaires under the supervision of a researcher. The demographic data collected included age, gender, ethnicity, marital status, employment, education, monthly income, and religion. The social data collected included self-perceived social support, diet, exercise, cigarette smoking, recreational drug use, and alcohol use. Clinical data were also obtained, including type of diabetes mellitus, duration of diabetes, number of medications, insulin therapy, self-perceived diabetic control, and comorbid hypertension, dyslipidaemia, renal disease, ischaemic heart disease, cancer, and cerebrovascular incident. The weight, height, and body mass index (BMI) of the participants were also obtained. A BMI of 18.5 to 24.95 was considered normal, 25.05 to 29.95 was considered overweight, and ≥30.05 was considered obese [13]. The clinical data collected were supplemented with information available from each participant's electronic medical record. This included blood test results processed in the ISO-certified laboratory of UKMMC, such as most recent haemoglobin A_1C_ (HbA_1C_), fasting blood glucose, blood urea nitrogen (BUN), creatinine, liver function tests, and lipid profile. An HbA_1C_ of ≥7.0% was used as an indicator of poor diabetic control [14]. In addition, four validated questionnaires were administered to participants. Depressive symptoms were assessed using a validated Malaysian version of the Beck Depression Inventory-II (BDI-II); anxiety symptoms were evaluated with the Generalized Anxiety Disorder-7 (GAD-7); personality traits were assessed with the Big Five Inventory (BFI); and quality of life was evaluated with the World Health Organization Quality of Life-BREF (WHOQOL-BREF).

#### 2.2.1. Generalized Anxiety Disorder-7 (GAD-7)

The GAD-7 is a self-rated screening tool and a severity indicator for generalized anxiety disorder (GAD) in the primary health care setting. It consists of seven items, each of which is rated on a 4-point Likert scale ranging from 0 (not at all) to 3 (nearly every day). As such, possible total scores range from 0 to 21, with higher scores indicating greater severity of GAD symptoms. The tool has a diagnostic cut-off point of 10, with a sensitivity of 89% and a specificity of 82% [15]. It has good psychometric properties with good internal consistency (Cronbach's *α* of 0.85) [16]. The Malay version of the GAD-7 has also demonstrated good psychometric properties with an acceptable internal consistency (Cronbach's *α* of 0.74) [17].

#### 2.2.2. Beck Depression Inventory-II (BDI-II)

The BDI was initially developed by Aaron Beck to assess cognitive symptoms of depression. It later incorporated somatic symptoms of depression to better reflect DSM criteria. The BDI-II is a self-administered questionnaire and consists of 21 items with total scores ranging from 0 to 63, with each item being scored from 0 to 3. Higher scores indicate greater severity of depressive symptoms. The BDI-II is reported to have excellent internal consistency with Cronbach's *α* of 0.90 [18]. The twenty-question Malay version of the BDI-II is also reported to have good psychometric properties, with good internal consistency (Cronbach's *α* of 0.80) [19].

#### 2.2.3. Big Five Inventory (BFI)

The BFI is a 44-item, self-rated tool used for assessing personality traits. The inventory's items are designed to measure five main domains: neuroticism (N; 8 items), openness to experience (O; 10 items), extraversion (E; 8 items), conscientiousness (C; 9 items), and agreeableness (A; 9 items). Each item is measured using a 5-point Likert scale, ranging from 1 (strongly disagree) to 5 (strongly agree). The BFI has good psychometric properties with good internal consistency (Cronbach's *α* average above 0.80) [20]. The Malay version of the BFI has also demonstrated good psychometric properties with good reliability (Hancock and Mueller's coefficient H ranging from 0.70 to 0.77 for all domains except for openness which had a coefficient H of 0.60, which may be a result of cultural differences in how openness is conceptualized) [21].

#### 2.2.4. The World Health Organization Quality of Life-BREF (WHOQOL-BREF)

The WHOQOL-BREF is a 26-item, self-rated tool used to evaluate the quality of life. The first two items assess the overall perception of QOL and overall perception of health, while the remaining 24 items measure specific domains of the quality of life: physical health QOL (7 items), psychological QOL (6 items), social QOL (3 items), and environmental QOL (8 items). Each item is scored with a 5-point Likert scale ranging from 1 (very poor) to 5 (very good). Three items (items 3, 4, and 26) are also negatively phrased, and hence, their scores are reversed when computing the total domain scores. The total score of each domain is then converted to a transformed score ranging from 4 to 20. The WHOQOL-BREF has good psychometric properties with internal consistency ranging from Cronbach's *α* of 0.55 to 0.89, and it has been found to be a valid and reliable alternative to the WHOQOL-100 for measuring QOL [22]. The Malay version of the WHOQOL-BREF has also demonstrated excellent psychometric properties with good internal consistency (Cronbach's *α* of 0.89) [23].

### 2.3. Statistical Analysis

All statistical analyses were performed using SPSS version 20 (SPSS Inc., Chicago, IL). Descriptive statistics were computed for demographic characteristics, lifestyle, social history, clinical factors, and the BFI, BDI-II, GAD-7, and WHOQOL-BREF scores. Categorical variables were described in frequency and percentage. Since the normality test indicated that the continuous variables were not normally distributed, these variables were reported in median and interquartile range (IQR). We first conducted the binary logistic regression analysis to evaluate the crude odds ratios (crude ORs) of the demographic characteristics, lifestyle behaviour, social history, clinical factors, and the BFI, BDI-II, GAD-7, and WHOQOL-BREF scores in predicting the outcome of diabetic control (good/poor). Then, variables with significant crude odds ratios (crude ORs, *p* < 0.05) were entered into a multivariate logistic regression model to assess the association of these variables with diabetic control by computing the adjusted odds ratios (adjusted ORs). Two-tailed tests were used, and a *p* value of <0.05 was considered statistically significant.

## 3. Results

### 3.1. Characteristics of Study Participants

The study recruited 300 participants, most of whom were in the elderly age group (median age = 63 years, IQR = 16 years). There were slightly more male participants (53%) than female (47%). Approximately two-thirds of the participants were Malay (65%), while the rest were either Chinese (18%) or another ethnic group (17%). The majority of participants were Muslim (66%), and a large proportion were married (77%). 84% of the participants completed at least a secondary education, although the majority of them were retired (72%). More than half (55.3%) of the participants reported living in a low-income household (monthly income < RM 3000), likely due to the high proportion of participants who were retired.

Most participants were nonsmokers (93%), did not consume alcohol (90%), had no history of illicit drug use (98%), and reported having good social support (80%). Most patients had type 2 diabetes (90%), with only a small minority having type 1 diabetes (7%) or gestational diabetes (2%). The median duration of diabetes was 14 years (IQR = 12 years). All patients were on oral hypoglycaemic medications, and most patients were on multiple medications (median number of medications taken = 6 types, IQR = 3 types). Nearly half (46%) of the patients were on insulin therapy. 69% of the participants had poor diabetic control (median HbA_1C_ = 7.6%, IQR = 2.7; median fasting blood glucose = 7.6 mmol/L, IQR = 2.7), although 71% of participants perceived that they had either *good* or *very good* diabetic management. Comorbid medical conditions were also common among participants, with 74% having hypertension, 51% having dyslipidaemia, 27% having ischaemic heart disease, 18% having renal disease, and 9% having previously experienced a cerebrovascular incident. The median BMI was 27.6 (IQR = 6.6), which was considered overweight. The demographic, social, and clinical characteristics of the participants are summarized in Table 1.

The BDI-II and GAD-7 results indicated that 20% of the participants had depression and 9% had anxiety. Assessment with the BFI revealed that the median domain scores were as follows: extraversion was 3.4 (IQR = 0.8), agreeableness was 3.8 (IQR = 0.4), conscientiousness was 3.7 (IQR = 0.6), neuroticism was 2.5 (IQR = 0.7), and openness to experience was 3.5 (IQR = 0.6). As for the WHOQOL-BREF scores, the median score for overall perception of QOL was 4.0 (IQR = 4.0), while the median score for overall perception of health was 3.0 (IQR = 3.0). The median score for physical health QOL was 14.3 (IQR = 3.4), psychological QOL was 15.3 (IQR = 2.7), social QOL was 16.0 (IQR = 2.7), and environmental QOL was 15.0 (IQR = 2.5). The psychological characteristics and QOL of the participants are summarized in Table 2.

### 3.2. Predictors of Poor Glycaemic Control

In unadjusted binary logistic regression analyses, the variables significantly associated with poor glycaemic control included the following: a secondary education or higher (crude OR = 2.33, 95% CI = 1.15–4.72), longer duration of diabetes mellitus (crude OR = 1.05, 95% CI = 1.02–1.08), taking three or more diabetic medications (crude OR = 3.11, 95% CI = 1.32–7.30), and greater number of days of missed medications (crude OR = 1.39, 95% CI = 1.11–1.73). On the contrary, older age (crude OR = 0.97, 95% CI = 0.95–0.99), absence of insulin therapy (crude OR = 0.32, 95% CI = 0.19–0.55), and better overall perception of QOL (crude OR = 0.60, 95% CI = 0.42–0.86) were protective against poor glycaemic control. None of the personality traits, psychiatric factors, or any other domains of QOL were associated with poor glycaemic control.

After adjusting for confounding variables in multivariate logistic regression analysis, there remained only two variables which predicted poor glycaemic control: longer duration of diabetes mellitus (adjusted OR = 1.11, 95% CI = 1.06–1.16) and greater number of days of missed medications (adjusted OR = 1.78, 95% CI = 1.21–2.61). In contrast, older age (adjusted OR = 0.95, 95% CI = 0.91–0.98) and better overall perception of QOL (adjusted OR = 0.37, 95% CI = 0.20–0.67) were protective against poor glycaemic control.  Table 3 summarizes the crude and adjusted ORs of the various factors in predicting poor glycaemic control.

## 4. Discussion

The aim of this study was to determine the prevalence of poor glycaemic control among Malaysian diabetic patients and its association with biopsychosocial factors, such as personality traits, psychiatric factors, and QOL. We found that 69% of patients had poor glycaemic control. This is similar to the prevalence reported by studies of diabetic patients in China, Ghana, Malaysia, and Egypt, which ranges from 50.3% to 74.3% [11, 24–26].

There were only two factors found to be predictive of poor glycaemic control: longer duration of diabetes mellitus, which increased the odds of poor glycaemic control by 1.1 fold, and greater number of days of missed medication, which increased the odds of poor glycaemic control by 1.8-fold. These findings support existing evidence from previous studies that suggest diabetic control worsens with increasing duration of diabetes mellitus [9, 24–27]. In fact, a duration of diabetes of ≥5 years has been shown to increase the risk of poor glycaemic control in type 2 diabetic patients [9, 27]. The median duration of diabetes in our study was 14 years. In patients with diabetes mellitus, the number and functioning of pancreatic *β*-cells deteriorate over time, leading to an increase in insulin resistance and a reduction in insulin secretion. This results in increasing difficulty in maintaining glycaemic control, which may explain why a longer duration of diabetes is associated with poorer glycaemic control [28]. The association between poor diabetic medication adherence and poor glycaemic control is consistent with two studies conducted in Tanzania and Ethiopia [29, 30]. It has been found that the pain and anxiety associated with daily insulin injections may increase the risk of insulin omission in diabetic patients [31, 32]. Since almost half of our participants were on insulin therapy (46%), this may have contributed to a lack of medication adherence and, thus, poor glycaemic control. In keeping with this, the absence of insulin therapy was shown to be an independent protective factor against poor glycaemic control in this study (crude OR = 0.32). Our findings highlight the importance of patient education and counselling in the management of diabetes mellitus, as a lack of understanding may lead to poor medication adherence. In order to encourage insulin adherence, patients need to appreciate the rationale for insulin therapy and the importance of regular follow-up for dose titrations.

Our study further identified two protective factors against poor glycaemic control: older age and a better overall perception of QOL. This first finding is consistent with several previous studies that have reported an association between older age and better glycaemic control [25, 33–36]. There is evidence that younger patients may be prescribed lower dosages of medication [33]. This could be a contributing factor as to why older patients have better glycaemic control compared to their younger counterparts. As far as we are aware, our study is the first to report that patients with better overall perception of QOL had an almost two thirds reduced odds of having poor glycaemic control. A strong sense of self-efficacy has been shown to be predictive of better self-care behaviours, better diabetic control, and greater QOL in patients with chronic illnesses [37, 38]. In our participants, self-efficacy may be a mediating factor in the relationship between perceived quality of life and glycaemic control. It would be beneficial to assess self-efficacy in this population in future studies.

Surprisingly, our findings demonstrated that psychiatric factors, namely, depression and anxiety, were not associated with glycaemic control. This contradicts the findings from two previous studies: a meta-analysis of 775 studies of diabetic patients and a literature review which indicated that depression predicts lower adherence to physical activity and dietary control and, hence, poorer glycaemic control [39, 40]. However, de Groot et al. reported that the effect size of depression in predicting poor glycaemic control is small, which may explain why depression was not a significant predictor of poor glycaemic control in our study. Conversely, our finding that anxiety is not predictive of poor glycaemic control is in line with the findings of a meta-analysis by Brown et al. in 2016 and a literature review by de Groot et al. in 2016.

Our findings also demonstrated that personality traits were not associated with poor glycaemic control. In the current literature, there exists mixed evidence regarding the relationship between personality traits and glycaemic control. In 2017, Novak et al. studied 117 couples with diabetes and demonstrated that neuroticism reduces dietary and exercise adherence and is therefore associated with lower diabetic efficacy [41]. Similarly, an Iranian study of type 2 diabetic patients reported neuroticism to be positively correlated with poor glycaemic control, while extraversion and conscientiousness were inversely correlated [42]. In addition, an Australian study of type 1 diabetic patients also reported that conscientiousness and agreeableness are significant predictors of good glycaemic control [43]. On the other hand, other studies specifically using the five-factor model of personality have found that there is no independent association between personality traits and glycaemic control [44, 45]. In line with these latter findings, we found that none of the big five personality traits were associated with glycaemic control. This discrepancy in the current literature may be a result of differences in the number of variables used in each study. For example, unlike our study, the first three aforementioned studies did not include demographic, social, and clinical factors as confounding factors. When demographic, social, and clinical factors are taken into account, personality traits do not appear to significantly predict glycaemic control.

Our findings need to be interpreted in light of a few limitations. The main limitation is the cross-sectional nature of the study, which prevents us from drawing any conclusions about causality between the various factors measured and glycaemic control. Second, multiple variables were tested for associations in this study, and this multiplicity may raise the possibility of false positives due to type 1 error [46]. While new strategies have been proposed to handle the issue of multiple testing [47], there are also strong arguments that it is not necessary to adjust for multiple comparisons in observational epidemiological studies [48]. In addition, this study was conducted in the setting of a tertiary healthcare centre based in Kuala Lumpur; therefore, the findings of this study may not be generalizable to the entire Malaysian diabetic population. We recommend that a prospective study with multicentre subject recruitment must be conducted in the future to confirm our findings. Finally, missing data may lead to a bias in the study findings; however, the proportion of missing data was small for most of the variables assessed except for monthly income (5.3% missing) and insulin therapy (16% missing).

Despite the limitations listed above, this study provides clinicians with comprehensive data on the biopsychosocial predictors of poor glycaemic control. These are important considerations when planning a multidisciplinary approach to diabetic management in the Malaysian context. To our knowledge, this is also the first study in Malaysia to evaluate the predictive effects of personality and quality of life on glycaemic control. It highlights the need for effective diabetes education in order to improve medication adherence, and it cautions healthcare providers to anticipate the negative effects associated with a longer disease duration. Our findings also emphasize the importance of assessing patients' perception of their QOL, as this appears to be associated with glycaemic control. A greater emphasis should be placed on evaluating the barriers to optimal glycaemic control, especially among younger patients. We hope that this study serves as a reminder for healthcare providers to evaluate glycaemic control in the greater context of each patient's life, as there are a number of biopsychosocial factors that may contribute to a patient's management of his or her diabetes.

## 5. Conclusion

In conclusion, the prevalence of poor glycaemic control in the Malaysian diabetic population is high. A longer duration of diabetes and poor medication adherence predict poor glycaemic control, while older age and better overall perception of QOL protect against poor glycaemic control. Psychological factors, namely, personality traits and psychiatric factors, are not associated with glycaemic control in the Malaysian diabetic population. The study findings emphasize the importance of the following: (1) psychosocial interventions such as patient education and counselling, (2) the need to strengthen treatment plan for patients with longer disease durations, (3) continued evaluation of patients' perception of their QOL, and (4) consideration of biopsychosocial factors, especially in younger patients, as part of the multidisciplinary approach to the management of patients with diabetes.

## Figures and Tables

**Table 1:** Demographic, social, and clinical characteristics of the participants.

Variables	Participants (*n*)	Percentage (%)
Age	63 years^a^	16^b^
Gender:		
Male	158	52.7
Female	141	47.0
Missing	1	0.3
Ethnicity:		
Malays	195	65.0
Non-Malays	105	35.0
Marital status:		
Married	231	77.0
Single/divorce/widow/widower	67	22.3
Missing	2	0.7
Education status:		
No education/primary education	45	15.0
Secondary education	133	44.3
Tertiary education	119	39.7
Missing	3	1.0
Employment status:		
Employed	80	26.7
Retired/unemployed	217	72.3
Missing	3	1.0
Monthly income:		
<Ringgit Malaysia (RM) 3,000	166	55.3
≥Ringgit Malaysia (RM) 3,000	118	39.3
Missing	16	5.3
Religion:		
Islam	199	66.3
Other religions	99	33.0
Missing	2	0.7
Cigarette smoking:		
Nonsmoker	280	93.3
Smoker	20	6.7
Alcohol use:		
Yes	26	8.7
No	271	90.3
Missing	3	1.0
Recreational drug use:		
Yes	5	1.7
No	294	98.0
Missing	1	0.3
Self-perceived social support:		
Poor	18	6.0
Moderate	48	16.0
Good	233	77.7
Missing	1	0.3
Types of diabetes mellitus:		
Gestational diabetes	6	2.0
Type 1 diabetes	22	7.3
Type 2 diabetes	269	89.7
Missing	3	1.0
HbA_1C_	7.6^a^	2.7^b^
Fasting blood glucose	7.1^a^	3.4^b^
Duration of diabetes mellitus	14 years^a^	12 years^b^
Number of medications taken	6 types^a^	3 types^b^
Number of diabetic medications taken	2 types^a^	1 type^b^
Insulin therapy:		
Yes	138	46.0
No	114	38.0
Missing	48	16.0
Frequency of clinic visits per year	2^a^	2^b^
Number of days of missed medications	0^a^	0^b^
Number of days of reduced medication intake	0^a^	0^b^
Self-perceived diabetic control:		
Poor	15	5.0
Moderate	70	23.3
Good	214	71.3
Missing	1	0.3
Diabetic control:		
Good (HbA_1C_ < 7.0%)	92	30.7
Poor (HbA_1C_ ≥ 7.0%)	208	69.3
Comorbid medical illnesses:		
Hypertension	222	74.0
Hypercholesterolemia	152	50.7
Ischaemic heart diseases	82	27.3
Cerebrovascular incidents	27	9.0
Renal diseases	53	17.7
Obesity status:		
No (body mass index < 25)	65	21.7
Overweight (body mass index between 25.0 and 29.9)	91	30.3
Obese (body mass index ≥ 30)	78	26.0
Missing	66	22.0
Body mass index (BMI)	27.6^a^	6.6^b^

^a^Median. ^b^Interquartile range (IQR).

**Table 2:** Psychological characteristics and quality of life of the participants.

Variables	Participants (*n*)	Percentage (%)
Anxiety disorders (assessed by the GAD-7):		
Yes	27	9.0
No	273	91.0
Depressive disorders (assessed by the BDI):		
Yes	60	20.0
No	240	80.0
Big Five Inventory (BFI) scores:		
Extraversion	3.4^a^	0.8^b^
Agreeableness	3.8^a^	0.4^b^
Conscientiousness	3.7^a^	0.6^b^
Neuroticism	2.5^a^	0.7^b^
Openness	3.3^a^	0.6^b^
WHOQOL-BREF scores:		
Overall perception of QOL	4.0^a^	4.0^b^
Overall perception of health	3.0^a^	3.0^b^
Physical health QOL	14.3^a^	3.4^b^
Psychological QOL	15.3^a^	2.7^b^
Social QOL	16.0^a^	2.7^b^
Environmental QOL	15.0^a^	2.5^b^

^a^Median. ^b^Interquartile range (IQR).

**Table 3:** Factors associated with poor glycaemic control among the participants.

Variables	Unadjusted OR (95% CI)	*p* value	Adjusted OR (95% CI)	*p* value
Age	0.97 (0.95-0.99)	0.002^∗^	0.95 (0.91-0.98)	0.002^∗^
Gender:				
Male	1			
Female	0.75 (0.46-1.22)	0.24	—	—
Ethnicity:				
Malays	1			
Non-Malays	1.13 (0.68-1.89)	0.64	—	—
Marital status:				
Married	1			
Single/divorce/widow/widower	1.72 (0.98-3.04)	0.59	—	—
Education status:				
No education/primary education	1	1		
Secondary education	2.33 (1.15-4.72)	0.019^∗^	1.73 (0.63-4.79)	0.29
Tertiary education	1.71 (0.84-3.44)	0.14	0.73 (0.24-2.19)	0.57
Employment status:				
Employed	1			
Unemployed/retired	1.22 (0.70-2.11)	0.48	—	—
Monthly income:				
<RM 3,000	1			
≥RM 3,000	1.43 (0.85-2.41)	0.18	—	—
Religion:				
Islam	1			
Other religions	1.03 (0.61-1.74)	0.91	—	—
Self-perceived social support:				
Poor	1			
Moderate	0 .44 (0.09-2.30)	0.33	—	—
Good	0.50 (0.11-2.36)	0.38	—	—
Cigarette smoking:				
Non-smoker	1			
Smoker	0.86 (0.30-2.49)	0.78	—	—
Alcohol use:				
No	1			
Yes	1.21 (0.52-2.82)	0.67	—	—
Recreational drug use:				
No	1			
Yes	0.56 (0.06-5.06)	0.60	—	—
Types of diabetes mellitus:				
Gestational diabetes	1			
Type 1 diabetes	2.00 (0.15-26.73)	0.60	—	—
Type 2 diabetes	0.42 (0.05-3.64)	0.43	—	—
Duration of diabetes mellitus	1.05 (1.02-1.08)	0.002^∗^	1.11 (1.06-1.16)	<0.001^∗^
Frequency of clinic visits	0.95 (0.86-1.05)	0.35	—	—
Comorbid hypertension:				
No	1			
Yes	0.99 (0.57-1.74)	0.98	—	—
Comorbid hypercholesterolemia:				
No	1			
Yes	1.03 (0.63-1.67)	0.92	—	—
Comorbid ischaemic heart diseases:				
No	1			
Yes	1.16 (0.67-1.99)	0.60	—	—
Comorbid cerebrovascular incident:				
No	1			
Yes	0.95 (0.40-2.25)	0.90	—	—
Comorbid renal diseases:				
No	1			
Yes	0.87 (0.45-1.68)	0.68	—	—
Number of diabetic medications:				
1 type	1	1		
2 types	1.73 (0.97-3.10)	0.064	1.10 (0.50-2.43)	0.82
3 types	3.11 (1.32-7.30)	0.009^∗^	1.60 (0.55-4.70)	0.39
4 types	5.12 (0.61-42.97)	0.13	0.90 (0.07-10.91)	0.93
Insulin therapy:				
Yes	1	1		
No	0.32 (0.19-0.55)	<0.001^∗^	0.52 (0.24-1.15)	0.11
Number of medications taken daily	1.00 (0.91-1.10)	0.96	—	—
Number of days of missed medication	1.39 (1.11-1.73)	0.004^∗^	1.78 (1.21-2.61)	0.003^∗^
Number of days of reduced medication intake	1.12 (0.97-1.28)	0.12	—	—
Self-perceived diabetic control:				
Poor	1			
Moderate	2.68 (0.77-9.38)	0.12	—	—
Good	0.91 (0.30-2.75)	0.86	—	—
Body mass index (BMI)	1.02 (0.96-1.07)	0.58	—	—
Obesity status:				
Not obese (BMI < 25)	1			
Overweight (BMI = 25-29.9)	1.02 (0.52-2.02)	0.95	—	—
Obese (BMI ≥ 30)	1.48 (0.71-3.09)	0.29	—	—
Anxiety disorders:				
No	1			
Yes	1.61 (0.62-4.13)	0.32	—	—
Depressive disorders:				
No	1			
Yes	1.42 (0.74-2.70)	0.29	—	—
Personality traits (BFI):				
Extraversion	1.16 (0.72-1.86)	0.55	—	—
Agreeableness	0.90 (0.51-1.60)	0.73	—	—
Conscientiousness	0.94 (0.55-1.59)	0.82	—	—
Neuroticism	1.15 (0.74-1.77)	0.54	—	—
Openness	1.27 (0.78-2.07)	0.35	—	—
Overall perception of QOL	0.60 (0.42-0.86)	0.005^∗^	0.37 (0.20-0.67)	0.001^∗^
Overall perception of health	0.83 (0.59-1.15)	0.26	—	—
WHOQOL-BREF scores:				
Physical health QOL	1.09 (0.99-1.21)	0.082	—	—
Psychological QOL	0.95 (0.84-1.06)	0.35	—	—
Social QOL	0.95 (0.86-1.05)	0.31	—	—
Environmental QOL	0.95 (0.85-1.07)	0.38	—	—

^∗^Statistical significance at *p* < 0.05.

## Data Availability

The data used to support the findings of this study is available upon request from the corresponding author.
